# Task-specific morphological and kinematic differences in Lipizzan horses

**DOI:** 10.3389/fvets.2025.1569067

**Published:** 2025-06-17

**Authors:** Manja Zupan Šemrov, Lucie Přibylová, Elena Gobbo

**Affiliations:** ^1^Department of Animal Science, Biotechnical Faculty, University of Ljubljana, Domžale, Slovenia; ^2^Department of Ethology and Companion Animal Science, Faculty of Agrobiology, Food, and Natural Resources, Czech University of Life Sciences Prague, Prague, Czechia

**Keywords:** equine, locomotion, non-pathological, morphology, positive experiences

## Abstract

**Introduction:**

Equine locomotion emerges from a dynamic interplay between morphology, biomechanics, and functional demands. This study examines the relationship between morphological measurements and gait kinematics in Lipizzan horses, a breed renowned for its diverse work tasks and standardized environmental conditions. These horses offer a unique opportunity to explore task-specific adaptations in biomechanics, with significant implications for breeding strategies and welfare practices.

**Materials and methods:**

The study involved 71 healthy Lipizzan horses that were housed at the Lipica stud farm and performed various work tasks. Morphological measurements were taken with the help of a sartorial meter and an equine measuring stick to determine head and body measurements. Both the left and right sides of the body were measured to ensure consistency. Kinematic data, including regularity, symmetry, cadence, dorsoventral power, propulsion power, stride length and speed, were recorded using the Equimetrix accelerometer at a sampling rate of 100 Hz. The data was collected during several walks and trots where the horses were led over a 50-meter track.

**Results:**

Task-based analysis revealed strong links between morphology and gait in four working groups, with distal limb measurements, especially hoof and pastern lengths, most consistently associated with stride and rhythm parameters. No significant associations were found at the whole-cohort level. Several morphological measurements showed contrasting effects across working groups, and half of the bilaterally measured traits revealed side-specific correlations. The clearest patterns emerged in horses used for general training and riding school. In horses in general training, strong associations were found between distal limb measurements and stride length or cadence, particularly during walk. In riding school horses, broader body measurements were linked to kinematic parameters including propulsion power, dorsoventral power, and symmetry.

**Discussion:**

This study highlights the dynamic interplay between conformation and functional demands in clinically sound horses. Rather than exerting fixed effects, morphological measurements interacted with work type to shape gait expression, even in the absence of pathology. These findings underscore the need to consider both structure and task when evaluating locomotion. Integrating morphometric assessment into training and selection strategies may support performance, soundness, and welfare in healthy working horses.

## 1 Introduction

The animal kingdom exhibits a remarkable diversity of locomotion (1), with each species using unique movement patterns tailored to its environment and needs (2). Quadrupedal mammals such as horses, for example, have fascinating and highly specialized locomotion, with the most common gaits being walk, trot, canter, and gallop (3). Each gait involves a distinctive patterns of leg movements and speeds that enable horses to move efficiently over different terrains. During walk, at least one front and one hind foot are always in contact with the ground, resulting in a consistent four-beat rhythm. In contrast, the trot is a two-beat gait in which all four feet lift off the ground, a characteristic that is also present in faster gaits such as the canter and gallop (4).

Gait is directly influenced by morphology, which serves as a structural foundation that both enables and limits the range of movement (5). Together, these two aspects show how the physical form of an organism influences the efficiency and effectiveness of its locomotion. Variations in bone length, joint structure, and muscle arrangement all play a crucial role in determining locomotor capabilities. Studies on mammals show that the length of the hind limbs and the ratio of the metatarsal to femur correlate with running speed (6). In humans, larger body size has been shown to correlate with higher optimal walking speed (7). Adaptations for speed are important in mammalian evolution, suggesting that animals optimize their morphology for speed and to reduce the cost of locomotion (8). In quadrupeds, the limbs are important to support the body mass, with the morphology of the forelimbs fulfilling various functional roles (9).

The locomotor system is particularly important in working animals, such as horses. In practice, selective breeding produced different types of horses - heavier draught horses were bred to pull heavy carts, while leaner and faster horses were selected for their speed and endurance (10). Also, parameters such as quality of gait, which reflects the way horses move according to functional and esthetic principles, are thought to predict future performance, making it an important breeding goal for European sport horses (11). Kinematic studies in horses have primarily focused on the limbs, as they are of immediate importance for performance, locomotor efficiency and the diagnosis of lameness. However, this does not mean that other regions of the body are irrelevant. For example, although gait speed does not differ significantly between breeds such as Andalusian, Arabian and Anglo-Arabian horses, marked differences have been found in terms of propulsion, timing of hoof contact, percentage of deceleration during stance and maximum limb retraction (12). These kinematic characteristics reflect different biomechanical strategies that influence how effectively horses generate thrust, absorb impact and coordinate their movement patterns, characteristics that are not only performance-related, but may also be relevant to breeding decisions and training programs (13–15). However, it remains unclear whether kinematics differ within a breed when horses perform different tasks and whether kinematic gait analysis can be reliably used to select horses for specific performances (13). However, some kinematic measurements are promising for breeding. For example, jump duration is a heritable variable that can be used as a breeding criterion in jumping horses (14), and trotter racehorses have a higher stride frequency and longer stance and propulsion durations at maximum speed (15), suggesting that targeted selection based on movement characteristics for specific disciplines is possible.

Kinematic data can be collected using marker-based video systems (16) or wearable inertial measurement units, such as EquiMoves (17) or Equimetrix (15, 18, 19), which incorporate accelerometers and gyroscopes to capture detailed motion parameters; Equimetrix was the system used in this study. Kinematic analysis has traditionally focused on pathological changes that limit locomotion, such as lameness and injury, primarily to prevent suffering and address negative welfare outcomes in horses (20–24). However, it is increasingly applied to investigate locomotion in clinically healthy horses across various disciplines. By observing the gait of healthy horses (without clinically observable locomotor abnormalities or other signs of disease), this study also fills a gap in the understanding of how non-pathological differences in kinematic parameters relate to morphology and type of work within a single breed. This within-breed focus allows us to explore both potentially inherited conformational traits that may have influenced selection for specific type of work, and physiological adaptations that may have developed over time through habitual training and exercise.

Accordingly, the aim of this study was to investigate how morphological and kinematic characteristics vary in Lipizzan horses performing different types of work under standardized environmental conditions to decipher possible selection- and experience-related influences on locomotion. The standardized breeding and housing conditions of Lipizzan horses at Lipica Stud Farm in Slovenia significantly reduce environmental variability. Their locomotion is fascinating due to their remarkable physical strength and speed (25, 26).

In recent years, the focus has shifted from simply managing negative experiences in farm animals to promoting environments in which animals can thrive (27, 28). Selecting horses with certain morphological and kinematic characteristics for appropriate types of work can provide animals with “a good life” by promoting skills such as competence (29) and resilience (30) and, thus, positive experiences. The implementation of such practices supports the concept of positive animal welfare, which is increasingly recognized as a more ethical approach to animal care. We hypothesize that certain morphological and kinematic characteristics can be identified depending on the type of work performed by the horse. These findings may help to develop future strategies for task selection, training and breeding, ultimately improving the welfare of working horses.

## 2 Materials and methods

### 2.1 Ethics statement

The procedures were approved by the Committee for the welfare of animals for experimental purposes of the Veterinary faculty of the University of Ljubljana under reference number 033-5/2024-5 as a part of a larger project. The designated authority in Lipica Stud Farm agreed on the procedures, as well as on the use of pictures, videos, and data for scientific purposes. The horse handlers/trainers were also given the right to withdraw from the study at any time and to be present during testing.

### 2.2 Animals and housing

This study examined a group of 71 healthy adult Lipizzan horses, consisting of 7 mares, 17 geldings, and 47 stallions, with ages ranging from 5 to 25 years (mean age: 10.1 ± 4.7 years). Horses showed no signs of lameness or disease prior or during data collection. All horses were sourced from the Lipica Stud Farm in Slovenia and housed in conventional individual boxes. Until the age of four, they were trained and ridden under the same conditions. After that point, they were assigned to different roles. Some were used for classical dressage, carriage pulling, or riding school activities. Others had not yet been assigned a specific task and continued with general daily training, such as lunging exercises. A few horses were not involved in any regular work or training at the time of the study (Table 1).

**Table 1:** The descriptions of working tasks and within-group demographics of horses.

Working task	Description	*N*	Mean age	Sex
Carriage pulling	Pulling the tourist carriages around Lipica premises	22	9.5 ± 3.0	6 mares, 7 geldings, 9 stallions
Classical dressage	Participation in classical dressage exhibitions and competitions	18	12.1 ± 5.1	2 geldings, 16 stallions
Riding school	Participation in touristic riding school lessons	8	11.5 ± 3.7	1 mare, 5 geldings, 2 stallions
General training	Involved in general everyday training, no specific working task	11	7.3 ± 1.8	1 gelding, 10 stallions
No working task	Not actively involved in everyday training or work	12	9.8 ± 7.6	2 geldings, 12 stallions

During their off-duty hours and overnight, all tested horses were kept in their individual boxes. Occasionally they were allowed access to pastures during the day. The horses had *ad libitum* access to fresh water and hay, and their primary diet comprised of individually tailored barley-oat mixture.

#### 2.3 Morphological measurements

**Figure 1:**
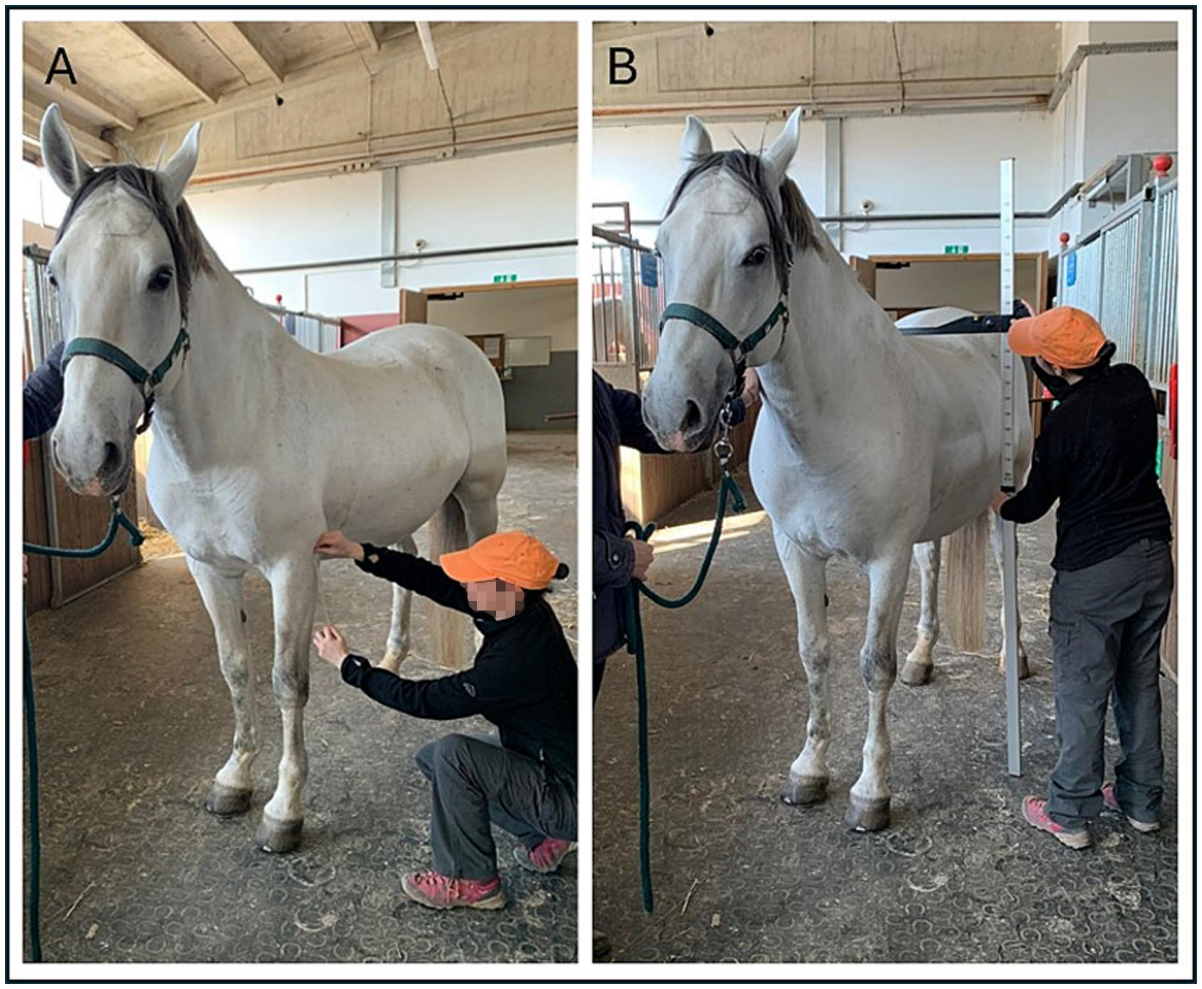
The use of a sartorial meter **(A)** and measuring stick **(B)** for morphological measurements.

**Figure 2:**
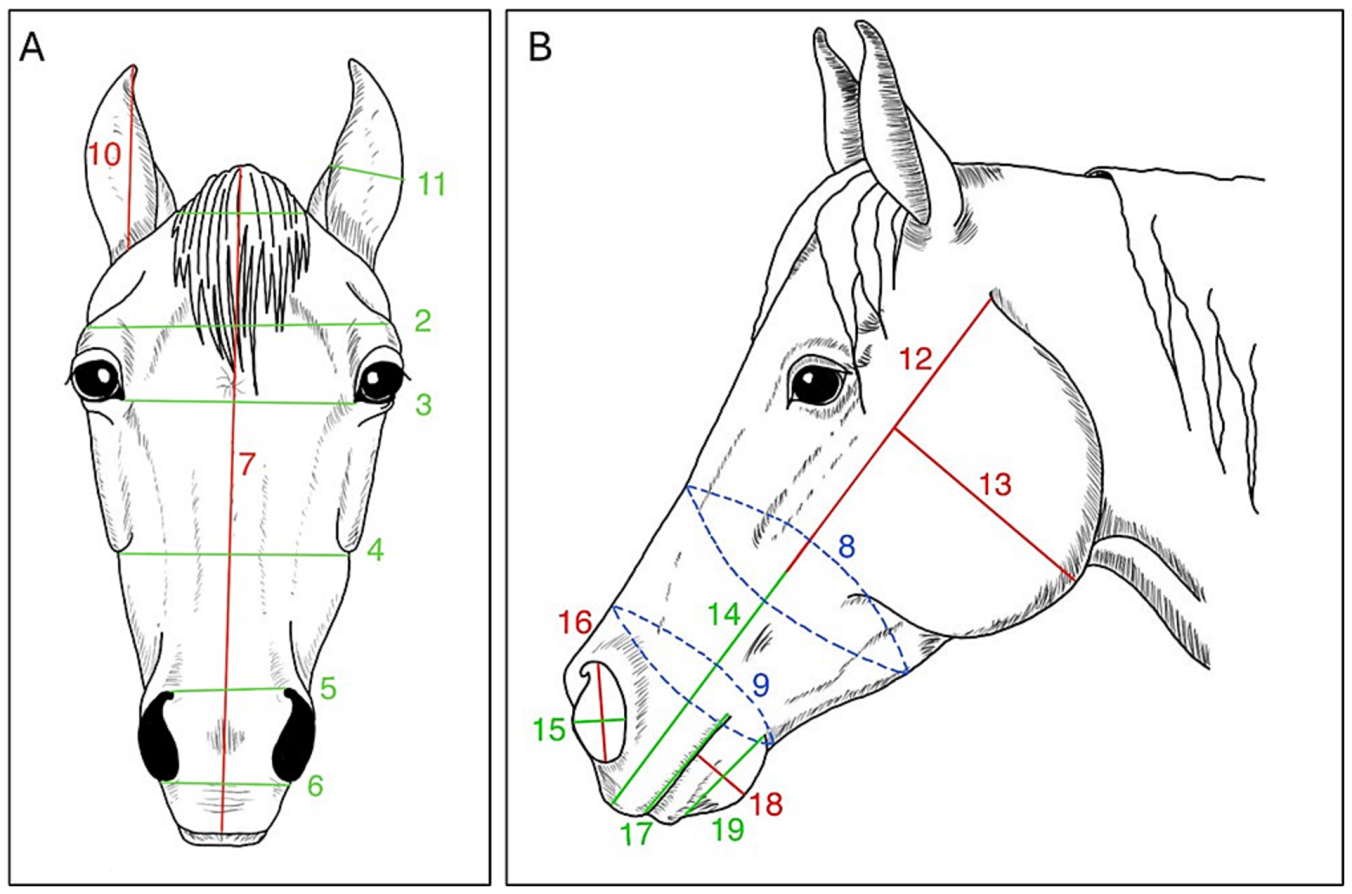
Head morphological measurements: **(A)** front view, **(B)** profile view.

**Figure 3:**
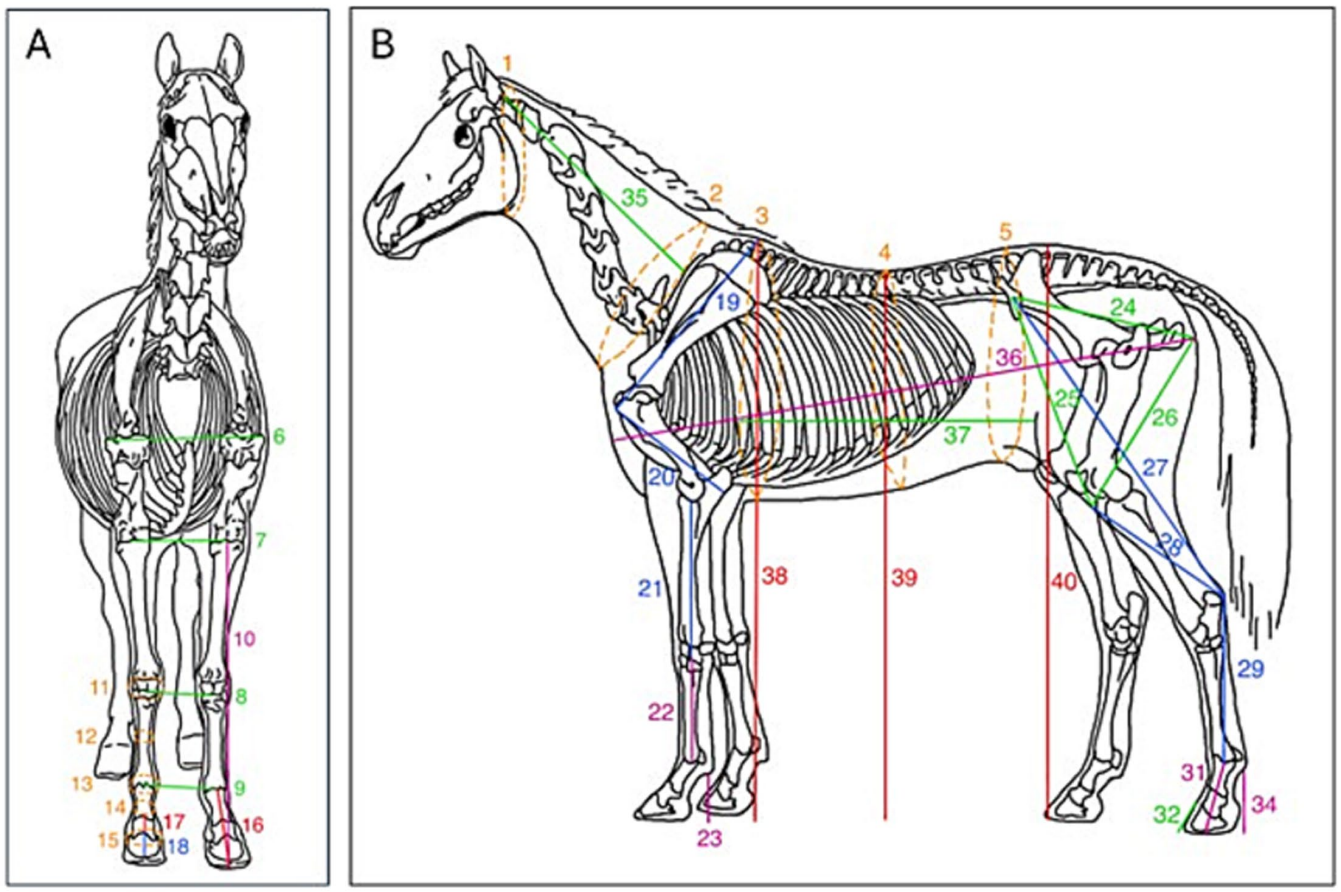
Body morphological measurements: **(A)** front view, **(B)** profile view.

Based on the Slovenian breeding program for Lipizzan horses, descriptions of morphological measurements (31) and previous study assessing morphology and other parameters in this breed (32), 95 different morphological measurements of the head and body were collected. Using a sartorial meter (Figure 1A), 29 measurements were taken from the front (Figure 2A), and both side profiles of the head (Figure 2B). Using the same approach, 63 measurements were taken from the front (Figure 3A) and both side profiles of the body (Figure 3B). The heights at the withers, back, and croup (Figure 3B) were taken from the left side of the horse using a specialized equine measuring stick (Figure 1B).

All measurements were performed with the horse standing on a flat surface (e.g., in front of the box) by a team of three experimenters - one responsible for handling the horse, one for taking the measurements, and one for recording the data. Initially, on 24 horses, the measurements were performed twice across two separate days by the same experimenter. Once the intra-class correlation coefficient (ICC) was calculated to assess consistency between the two sets of measurements and demonstrated excellent reliability (ICC = 0.99), subsequent measurements for the remaining horses were conducted only once.

From the body measurements, the horse’s weight was calculated using a formula: Weight = (girth^2^ * length)/Y. Girth was defined as body circumference behind the elbow and just behind the highest point of the withers (in cm), while the length was defined as the distance from the greater tubercle of the humerus to the tuber ischia (in cm). The used value for Y (in cm^3^/kg) was 11,877, since Carroll and Huntington (33) presented it as the most accurate value.

#### 2.4 Kinematic recordings

**Figure 4:**
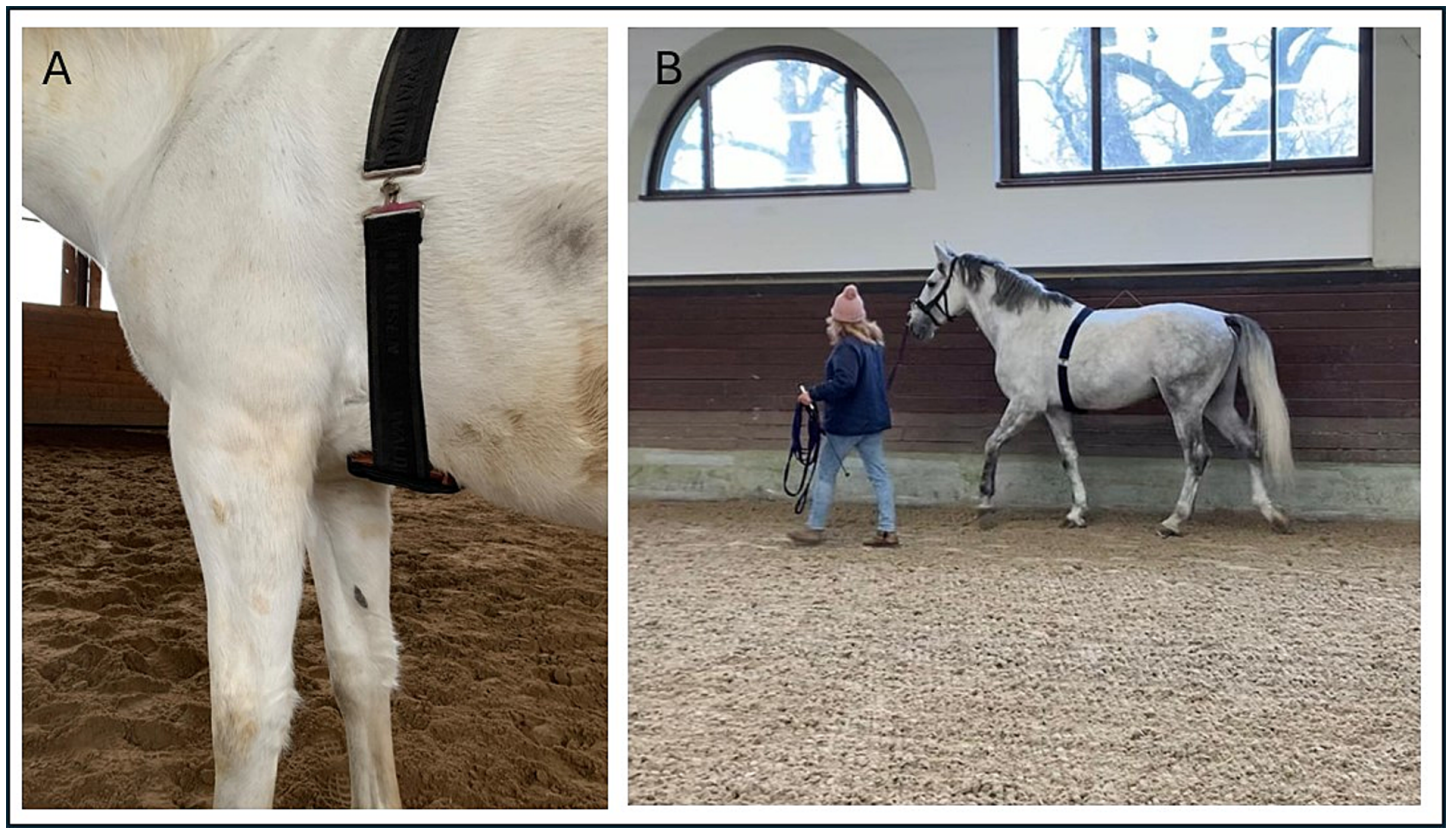
The application of the device: **(A)** placement of the device, **(B)** example of kinematic assessment during walk.

The kinematic recordings were performed using Equimetrix®, a three-dimensional accelerometer known for its validity and reproducibility (15, 19). This device consisted of an acceleration sensor and a data logger enclosed in a small block (4 × 2.2 × 1.7 cm). The block was placed in a leather bag and secured with an elastic strap to the caudal part of the sternum at the level of the chest girth (Figure 4A). The data were recorded continuously at a sampling rate of 100 Hz. As previously proposed, the horse was led by the hand (20) and covered a distance of 50 meters at its own comfortable speed (34), first at a walk, then at a trot–each activity was repeated four times (Figure 4B). The surface (freshly leveled sandy ground) was the same for all horses. One experimenter was responsible for handling the horse, while the other monitored the time with a stopwatch (for manual calculation of speed). This process was repeated twice within a week, with the same experimenter positioning the device and leading the horse. The data were uploaded to the Equimetrix Centaure® software for processing. The kinematic parameters analyzed automatically [for description of calculations see Leleu et al. (15, 19)] are described in Table 2. The mean values of all walk and trot repetitions were used for statistical analysis.

**Table 2:** The descriptions of kinematics parameters.

Parameter	Unit	Description
Speed	m/s	The rate at which the horse covered the distance of 50 m
Stride length	m	Distance covered between successive placements of the same hoof
Cadence	strides/s	The number of strides taken in 1 s
Regularity	dimensionless	The consistency of successive stride acceleration patterns.
Symmetry	dimensionless	The similarity between the acceleration patterns of the right and left diagonals
Dorsoventral power	g^2^/Hz	Loading activity and limb suspension.
Propulsion power	g^2^/Hz	Acceleration and deceleration along the longitudinal axis.

#### 2.5 Statistical design and analyses

We conducted a correlation analysis using the SAS software package (Statistical Analysis System, version 9.4) to explore the relationships among various phenotypic measurements in horses engaged in different working tasks. We employed Pearson’s correlation coefficient, calculated using the CORR procedure. In the first step of the analysis, we reduced the dataset by excluding morphological measurements that were highly intercorrelated (r ≥ 0.8). When measurementsfrom both sides of the body were strongly correlated, we retained the left-sided measurements and excluded the right-sided ones to avoid redundancy. Additionally, since all three height measurements and distances between the fetlocks and the carpi were strongly correlated, we retained only the height at the withers (FB38) and the distance between the carpi (FB08). This reduction resulted in a final set of 63 morphological measurements. In the second step, to assess the strength and direction of linear relationships between variables, we used partial correlation with PARTIAL statement to control for speed and age. For clarity, only results that met the criteria of a strong (r ≥ 0.8) or a very strong correlation (r ≥ 0.9) and a significance level below 0.025, considering Bonferroni correction for multiple testing, were included in the results section. Our analysis uncovered several significant correlations, highlighting the importance of these variables and laying the groundwork for further exploration of their interdependencies. To quantify within-group variability and support interpretation of correlation results, group-specific means and standard deviations (SDs) were calculated. Additionally, thresholds at ± 2 SD and ± 3 SD from the group mean were used to identify potential outliers. Scatterplots were generated separately for each group to visualize the distribution of data points and assess the consistency of associations, as well as to identify potential outliers.

Recognizing that a horse’s body weight could influence the observed correlations, we applied the General Linear Model (GLM) procedure to examine the effect of different work tasks on weight. To gain deeper insights into the differences among the levels of that variable, we performed pairwise comparisons using Tukey’s adjustment method to control for Type I error, thus preserving confidence levels across multiple comparisons. Ultimately, we found no statistically significant effect. One-way analysis of variance was used to assess differences in weight, kinematic parameters and the most important morphological measurements among the five working groups of horses. It was followed by Tukey’s *post-hoc* test to identify pairwise group differences. Considering Bonferroni correction for multiple testing, values below 0.025 were considered significant.

### 3 Results

#### 3.1 All horses

Table 3 presents the descriptive statistics of the kinematic parameters for a horse’s walk and trot, highlighting the numerical differences due to the distinct nature of these gaits. The correlation analysis between gait kinematics and morphological characteristics for both walk and trot, conducted across all horses (*n* = 71), revealed only weak associations (correlation coefficients < 0. 44). Due to a large number of results (*n* = 756), these correlations are not included in the text.

**Table 3:** Mean values (± standard deviations) of kinematic parameters.

Horses	Gait	Regularity (dimensionless)	Symmetry (dimensionless)	Cadence (stride/s)	Dorso-ventral power (g^2^/Hz)	Propulsion power (g^2^/Hz)	Stride length (m)	Speed (m/s)
All horses	Walk	155.70 ± 37.81	192.72 ± 40.55	0.94 ± 3.24	0.52 ± 0.23	0.97 ± 0.44	1.78 ± 0.15	1.42 ± 0.11
	Trot	308.51 ± 44.17	259.59 ± 42.59	1.29 ± 0.06	14.98 ± 3.45	4.87 ± 1.86	2.45 ± 0.26	3.18 ± 0.40
General training	Walk	145.70 ± 28.75	180.94 ± 39.42	0.79 ± 0.06	0.51 ± 0.19	0.85 ± 0.27	1.78 ± 0.07	1.42 ± 0.11
	Trot	307.80 ± 44.29	251.15 ± 35.42	1.29 ± 0.04	15.19 ± 2.76	4.05 ± 1.11	2.36 ± 0.19	3.03 ± 0.25
No work	Walk	146.13 ± 46.24	194.72 ± 42.78	0.84 ± 0.12	0.52 ± 0.25	0.88 ± 0.29	1.72 ± 0.21	1.44 ± 0.12
	Trot	308.78 ± 48.06	259.60 ± 38.49	1.32 ± 0.07	15.50 ± 3.38	4.22 ± 1.13	2.43 ± 0.26	3.21 ± 0.40
Classical dressage	Walk	167.41 ± 38.65	185.02 ± 36.59	0.83 ± 0.13	0.59 ± 0.22	1.11 ± 0.49	1.77 ± 0.15	1.44 ± 0.12
	Trot	316. 13 ± 40.52	252.74 ± 43.79	1.27 ± 0.05	14.04 ± 3.62	4.57 ± 1.64	2.40 ± 0.25	3.06 ± 0.36
Riding school	Walk	166.03 ± 36.46	195.56 ± 39.34	0.79 ± 0.05	0.55 ± 0.30	0.95 ± 0.39	1.77 ± 0.11	1.40 ± 0.15
	Trot	310.19 ± 41.72	273.48 ± 47.09	1.32 ± 0.05	14.26 ± 0.05	6.03 ± 2.02	2.41 ± 0.15	3.19 ± 0.23
Carriage pulling	Walk	152.57 ± 33.15	202.80 ± 41.04	0.77 ± 0.18	0.47 ± 0.18	0.98 ± 0.51	1.83 ± 0.13	1.39 ± 0.10
	Trot	301.86 ± 45.07	264.36 ± 43.78	1.29 ± 0.07	15.64 ± 3.68	5.47 ± 2.17	2.57 ± 0.30	3.34 ± 0.48

#### 3.2 Horses grouped by type of work

After grouping the horses based on five different working tasks, 3 head measurements and 15 body measurements (Table 4) showed very strong (≥ 0.8) and excellent (≥ 0.9) correlations with kinematic parameters in four groups. Of the 18 significantly associated measurements, 14 were measured bilaterally, and half of these showed side-specific correlations. There was no statistical difference between the type of work they performed and their weight (*p* = 0.35; *F* = 1.13).

**Table 4:** The descriptions of all morphological measurements that correlate with kinematic parameters and working group they appear in.

Measurement name	Description	Working group	Figure
Head (FH)			
FH04	Distance between the endpoints of facial crest bones	2	Figure 2A
FH06	Distance between the endpoints of nostrils	4	Figure 2A
FH14	Distance between the end of facial crest bone and the end of the muzzle	4	Figure 2B
Body (FB)			
FB01	Upper part of the neck circumference	4	Figure 3B
FB02	Lower part of the neck circumference	1,4	Figure 3B
FB03	Body circumference at the wither	4	Figure 3B
FB07	Distance between the elbows	4	Figure 3A
FB08	Distance between the left and right carpus on forelegs	2	Figure 3A
FB12	Cannon bone circumference	4	Figure 3A
FB14	Pastern circumference on forelegs	4	Figure 3A
FB16	Distance between the fetlock joint and the end of the forehoof	4	Figure 3A
FB17	Fore pastern length	1,2,4	Figure 3A
FB18	Forehoof length	1,2,4	Figure 3A
FB24	Distance between the tuber coxae and tuber ishii	4	Figure 3B
FB27	Distance between the tuber coxae and the point of hock	4	Figure 3B
FB28	Distance between the stifle joint and the point of the hock	4	Figure 3B
FB32	Hind hoof length	1	Figure 3B
FB36	Body length	3,4	Figure 3B

Working group: 1 = general training, 2 = no working task, 3 = classical dressage, 4 = riding school.

##### 3.2.1 General training

For horses in general training, three very strong and three excellent correlations were found during walk (Table 5). Lower part of the neck circumference was negatively correlated with regularity. Length of the pastern on forelegs was negatively correlated with cadence and positively with stride length. Forehoof length was positively correlated with symmetry. Hind hoof length was positively correlated with stride length and negatively with cadence, the latter showing the only correlation with a coefficient exceeding 0.9 and the lowest *p*-value observed across all measurements, indicating an exceptionally strong and statistically significant relationship. During trot, there were no correlations found.

**Table 5:** Correlations for horses from different working groups between body measurements and kinematics during walk and trot.

Working group	Gait	Body measurement	Regularity	Symmetry	Cadence	Dorsoventral power	Propulsion power	Stride length
General training	Walk	FB02	−0.85**					
		FB17R			−0.88**			0.91**
		FB18LR		0.91**				
		FB32R			−0.92***			0.82**
No working task	Walk	FB08			0.84**			−0.87**
	Trot	FB17R	0.87**					
		FB18LR			0.86**			−0.85**
		FH04					−0.83**	
Classical dressage	Walk	FB36LR						−0.81***
Riding school	Walk	FB02				0.87*		
		FB03				0.95**		
		FB07						0.87*
		FH14LR		−0.87*			0.92**	
		FB17R					0.89*	
		FB18LR					0.95**	
		FB24LR				0.89*		
		FB27LR					0.88*	
		FB28LR					−0.90*	
		FB36LR	−0.92**					
	Trot	FB01		−0.93**				
		FB07						0.88*
		FB12R				0.91*		
		FB14L				0.91*		
		FB16R					0.87*	
		FH06	0.89*					

*p < 0.025; **p < 0.005; ***p < 0.0005. L – left side of the body; R – right side of the body.

##### 3.2.2 No working task

For horses with no working tasks, two very strong correlations were found during walk (Table 5). Distance between the left and right carpus on forelegs was positively correlated with cadence and negatively with stride length. During trot, four very strong correlations were found (Table 5). Length of the pastern on forelegs was positively correlated with regularity. Forehoof length was positively correlated with cadence and negatively with stride length. Distance between the endpoints of facial crest bones was negatively correlated with propulsion power.

##### 3.2.3 Classical dressage

For horses in classical dressage, one very strong correlation was found during walk (Table 5). Body length was negatively correlated with stride length. During trot there were no correlations found.

##### 3.2.4 Riding school

For horses used in riding school, six very strong, and five excellent correlations were found during walk (Table 5). Lower part of the neck and body circumferences were positively correlated with dorsoventral power. Distance between the elbows was positively correlated with stride length. Pastern circumference on forelegs was negatively correlated with symmetry and positively with propulsion power. Length of the fore pastern and forehoof and distance between the tuber coxae and the point of hock were positively correlated with propulsion power, while the distance between the stifle joint and the point of the hock was negatively correlated. Distance between the tuber coxae and the tuber ishii was positively correlated with dorsoventral power. Body length was negatively correlated with regularity.

During trot, three very strong, and three excellent correlations were found (Table 5). Upper part of the neck circumference was negatively correlated with symmetry. Distance between the elbows was positively correlated with stride length. Cannon bone and pastern circumference on forelegs were positively correlated with dorsoventral power. Distance between the fetlock joint and the end forehoof was positively correlated with propulsion power. Distance between the endpoints of nostrils was negatively correlated with regularity.

##### 3.2.5 Carriage pulling

For horses used for carriage pulling, there were no correlations found during walk or trot.

##### 3.2.6 Overlap between working groups

Several morphological measurements were found to be correlated with kinematic measurements in two or more working groups (Table 4). For example, the lower neck circumference showed opposite relationships in different groups—it was negatively correlated with regularity in group 1 but positively correlated with dorsoventral power in group 4. Body length was negatively associated with stride length in group 3 and with regularity in group 4. The length of the foreleg pastern displayed multiple correlations: in group 1, it was negatively correlated with cadence and positively with stride length; in group 2, it was positively associated with regularity; and in group 4, with propulsion power. The length of the fore hoof was positively correlated with symmetry in group 1 and with propulsion power in group 4. In group 2, it showed a positive correlation with cadence and a negative one with stride length.

##### 3.2.7 Within working groups variability

A total of 14 individual data points were identified as outliers based on the ± 2 standard deviation (SD) criterion, whereas no outliers were detected using the ± 3 SD threshold (Supplementary File S1). Of these, six corresponded to kinematic parameters and eight to morphological measurements. The identified outliers originated from nine individual horses. Three of these, each representing a different work group, exhibited two or three outliers across different measurements. The highest number of outliers (*n* = 4) was identified in the riding school group, which represented the smallest working group in the study.

**Figure 5:**
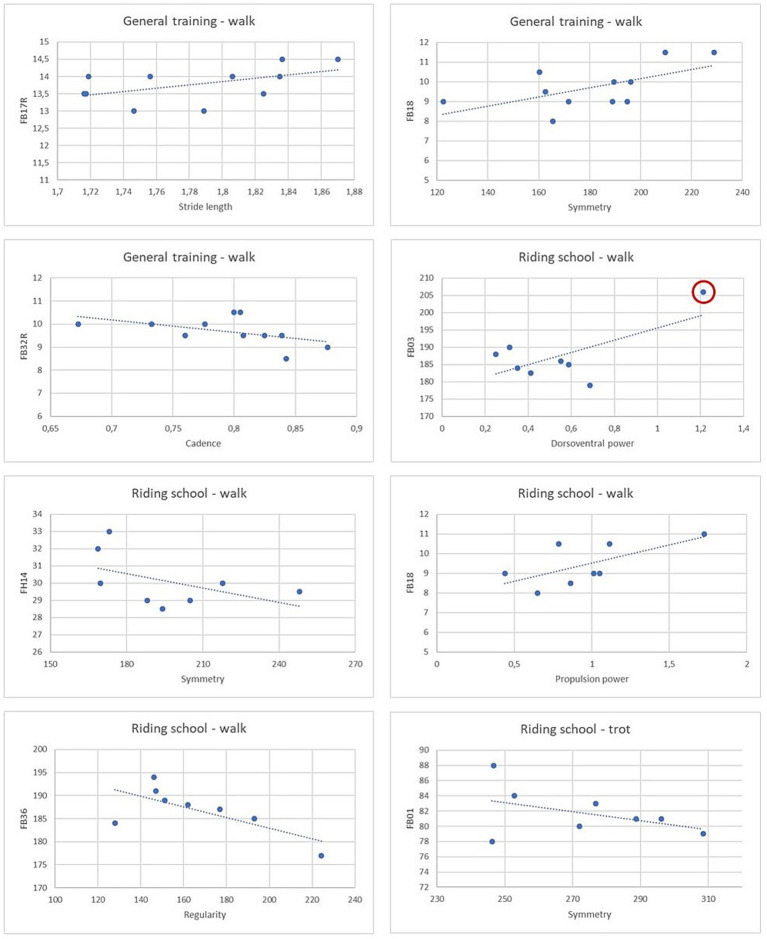
Scatterplots of the eight strongest correlations. A detected outlier is marked in red.

Scatterplots of the eight strongest correlations (r > 0.9, *p* < 0.0001) are presented in Figure 5, while additional scatterplots for important correlations are provided in Supplementary File S1. Among these strongest correlations, only a single outlier was identified and observed in the riding school group during walk, for body circumference at the withers (FB03; Figure 5).

#### 3.3 Differences between working groups

No statistically significant differences between working groups were observed in kinematic parameters during walk (Supplementary Table S1). During trot, only one parameter, propulsion power, showed a statistically significant difference (Supplementary Table S1). *Post-hoc* analysis indicated a trend toward a difference between horses in general training and those used in riding schools, though this did not reach statistical significance (*p* = 0.082).

Among the 18 most important morphological measurements, statistically significant differences were found in two: lower part of the neck circumference (FB02) and right hind hoof length (FB32R) (Supplementary Table S2). For FB02, *post-hoc* analysis revealed a significant difference between carriage-pulling horses and those used in classical dressage (*p* = 0.011). For FB32R, *post-hoc* analysis revealed a significant difference between carriage-pulling horses and those with no working task (*p* = 0.002). There were no statistically significant differences in weight.

Using multiple criteria to assess the meaningfulness of the observed correlations, all of the most statistically significant associations were also determined to be biologically meaningful. The task-specific correlations appear particularly informative, as they were minimally affected by outliers.

### 4 Discussion

In this study, we investigated the relationship between morphological measurements and gait kinematics in Lipizzan horses, a breed well suited for biomechanical analysis due to its involvement in a range of working tasks. This study focused exclusively on non-pathological, functionally sound gaits. It contributes to a broader understanding of equine locomotion in routine working environments, an area that remains underrepresented in a literature that largely focuses on lameness and injury detection. When the population was assessed as a whole, no significant correlations were detected between morphological measurements and kinematic parameters. This likely reflects the heterogeneity introduced by combining horses with different functional demands and movement patterns. Once horses were grouped by type of work, several clear and group-specific associations emerged, highlighting the task-dependent nature of conformation–locomotion relationships. These findings align with previous work demonstrating complex interactions between genetics, morphology, and kinematic measurements (35). Moreover, the near absence of statistically significant differences in both kinematic and morphological measurements between groups, as well as a small number of detected outliers, further supports the importance of task-specific associations between morphology and kinematics.

The clearest task-specific associations were observed in the general training group. Several notable correlations between morphological measurements and kinematic parameters appeared, particularly during walk. The strongest association was the negative relationship between hind hoof length and cadence, suggesting that horses with longer hind hooves take fewer steps. This may reflect a biomechanical trade-off, in which longer hooves prolong the break over phase and increase distal limb mass, resulting in reduced stride frequency (36, 37). Simultaneously, hind hoof length was positively associated with stride length, indicating a compensatory mechanism that may help maintain forward progression. A similar pattern emerged for fore pastern length, which was positively associated with stride length. While prior studies have focused on pastern angle (38), our linear measurements support the view that longer pasterns contribute to extended stride mechanics. Together, these findings suggest that distal limb conformation plays a meaningful role in shaping gait characteristics during general training activities.

In addition to the general training group, the riding school horses also showed numerous correlations, particularly during walk, where all measured kinematic parameters, except cadence, were affected. This extensive pattern may reflect the highly variable movement demands of riding school environments, where horses must frequently adapt to changes in rider ability, balance, and movement cues (39). Such variability may amplify the expression of morphology-dependent gait patterns. Interestingly, narrower nostril width was associated with lower regularity, suggesting a potential link between craniofacial measurements and coordination, although this warrants further investigation. However, the unexpectedly high number of correlations in this small group calls for cautious interpretation. Small sample sizes increase the influence of individual variability, potentially inflating correlation strength and limiting generalizability. Future studies with larger cohorts are needed to confirm the robustness of these associations.

In contrast to these findings, the classical dressage group exhibited only one significant correlation. Longer body length was associated with shorter stride length. Although limited, this finding is relevant for dressage, where extended, expressive strides are central to performance (40). It suggests that body length may influence a horse’s capacity to achieve the desired movement profile in this discipline and should be considered during selection and training.

The group of horses without assigned working tasks showed a different set of correlations. A shorter distance between the carpal joints was associated with increased cadence and reduced stride length during walk. This finding aligns with previous observations linking narrower limb spacing to more vertical, collected movement patterns, which support increased cadence but limit protraction and stride length (41). In trot, longer forehoof length was associated with increased cadence and reduced stride length. This is the opposite of what was observed in the general training group, where longer hind hooves were linked to decreased cadence and increased stride length. These contrasting patterns may reflect anatomical differences between fore- and hind hooves. As noted by Tijssen et al. (42), hind hooves typically have a steeper dorsal wall angle and are narrower than forehooves. Such differences can influence the hoof unrollment pattern and, in turn, the dynamics of limb movement. This suggests that the absence of structured training may alter how morphological measurements are expressed in locomotion. Additionally, longer fore pasterns were associated with greater regularity, a finding not observed in other groups. Narrower facial crest width was also linked to increased propulsion power in this group, though the mechanism remains unclear and warrants further study.

The carriage-pulling group, despite being the largest, showed no significant correlations. The linear, repetitive nature of carriage work likely contributes to highly uniform gait patterns, reducing inter-individual variability and masking the effects of morphological variation. Furthermore, the mechanical restrictions of harness and carriage equipment may limit natural variation in limb movement, diminishing the expression of conformation-dependent gait differences.

Despite variation across working groups, several morphological measurements, particularly forelimb hoof and pastern lengths, emerged repeatedly as important predictors of gait kinematics. However, these associations differed by context. The same measurement was linked to different kinematic parameters depending on the horse’s work. This reinforces the idea that morphological measurements do not exert a fixed influence, but rather interact dynamically with training, task demands, and environmental context. Such findings underscore the value of aligning selection, management, and conditioning strategies with both morphological structure and functional use.

Beyond work-specific patterns, the analysis revealed consistent bilateral symmetry in many morphological–kinematic associations. Among the measurements significantly correlated with movement parameters and measured on both sides of the body, half showed bilateral associations, while the rest were side-specific. This suggests that asymmetries may develop in response to specific work demands or individual adaptation. For example, dressage often involves lateralized, non-natural movements that can promote asymmetry (43), while variability in rider input or saddle fit may further contribute to uneven loading (44). In contrast, carriage-pulling typically involves linear, symmetrical movement with minimal lateral bias. These asymmetries may reflect biomechanical adaptations or training-induced preferences. Nonetheless, the predominance of bilateral correlations supports the established importance of coordinated limb function for maintaining gait stability and reducing injury risk (21, 34, 45).

Although no significant differences were observed between working groups, several strong correlations were detected within groups. This pattern suggests that individual-level variation, rather than group-level context, underlies the observed measurements relationships. The absence of outliers in these associations further supports the robustness of the findings and indicates that the correlations reflect biologically meaningful patterns rather than statistical artefacts. These within-group associations may point to stable, trait-level mechanisms that are not captured by comparing group means alone, highlighting the importance of individual differences in animal research.

One methodological limitation of this study was the inability to control for sex distribution across working groups. Although previous studies have reported sex-related differences in equine gait and performance traits, findings remain inconsistent (46–49). Management practices often favor geldings for riding school tasks due to their predictability, and stallions for high-performance work, such as classical dressage, due to their strength and expression (49, 50). These trends were reflected in our sample, with mares underrepresented in physically demanding work. While sex may influence movement to some extent, the group-specific associations observed in our data appear consistent across sexes.

Despite this limitation, understanding how conformation relates to gait can inform individualized training intensity, conditioning programs, and the selection of horses for specific tasks, particularly when supported by biomechanical analysis of horse–rider interaction (51). For example, prioritizing horses with favorable morphometric profiles may enhance movement efficiency, reduce strain on vulnerable structures, and support long-term soundness. Aligning morphology with functional demands not only promotes biomechanical efficiency and resilience but, in line with the principles of positive animal welfare (52), by supporting horses that are physically capable, adaptable, and confident in their roles.

### 5 Conclusion

This study demonstrated that the relationship between morphological measurements and gait kinematics in Lipizzan horses is strongly influenced by the type of work performed. While no significant associations were detected across the entire cohort, task-specific analyses revealed clear and meaningful correlations within individual working groups. Furthermore, the minimal statistically significant differences between groups, combined with the small number of outliers, highlight the importance of assessing these associations within specific work contexts rather than across a heterogeneous population. The most pronounced associations involved distal limb measurements, particularly hoof and pastern lengths, which were linked to stride length, cadence, and regularity. The observed effects were most evident in horses engaged in general training and riding school activities. Importantly, the same morphological measurements influenced different kinematic parameters depending on the work context, underscoring the dynamic interaction between conformation and functional demands. These findings highlight the practical value of morphometric assessment in supporting informed training decisions, improving performance and soundness, and aligning horses more effectively with the physical demands of their intended work.

## Data Availability

The original contributions presented in the study are included in the article/Supplementary material, further inquiries can be directed to the corresponding author/s.
