# Supplementary material for: Task-specific morphological and kinematic differences in Lipizzan horses
Source: Front Vet Sci. 2025 Jun 17;12:1569067. doi: 10.3389/fvets.2025.1569067 (PMC12208837; doi:10.3389/fvets.2025.1569067)
Supplement: Supplementary file 2 [file Table_2.docx]

Supplementary Material

Supplementary Table S1. Comparison of kinematic parameters between horse groups during walk and trot.

| Gait | Parameter | F-value | P-value |
| --- | --- | --- | --- |
| Walk | Regularity | 1.44 | 0.23 |
|  | Symmetry | 1.64 | 0.18 |
|  | Cadence | 1.42 | 0.79 |
|  | Dorsoventral power | 0.95 | 0.44 |
|  | Propulsion power | 0.97 | 0.43 |
|  | Stride length | 1.41 | 0.24 |
|  | Speed | 0.63 | 0.64 |
| Trot | Regularity | 0.49 | 0.74 |
|  | Symmetry | 1.94 | 0.11 |
|  | Cadence | 1.62 | 0.18 |
|  | Dorsoventral power | 0.84 | 0.51 |
|  | Propulsion power | 3.03 | 0.023 |
|  | Stride length | 2.34 | 0.06 |
|  | Speed | 2.51 | 0.05 |

Supplementary Table S2. Group differences in the most important morphological features, including Mean ± SD values.

| Feature | Group | Mean ± SD | F-value | P-value |
| --- | --- | --- | --- | --- |
| FH04 | General training | 29.64 ± 1.36 | 0.26 | 0.90 |
|  | No working task | 29.71 ± 1.72 |  |  |
|  | Classical dressage | 29.31 ± 0.89 |  |  |
|  | Riding school | 29.69± 1.46 |  |  |
|  | Carriage pulling | 29.43 ± 1.25 |  |  |
| FH06 | General training | 6.05 ± 0.27 | 0.40 | 0.81 |
|  | No working task | 6.13 ± 0.74 |  |  |
|  | Classical dressage | 6.19 ± 0.46 |  |  |
|  | Riding school | 6.25 ± 0.65 |  |  |
|  | Carriage pulling | 6.05 ± 0.43 |  |  |
| FH14 | General training | 29.14 ± 1.34 | 1.75 | 0.15 |
|  | No working task | 29.58 ± 1.16 |  |  |
|  | Classical dressage | 29.14 ± 0.97 |  |  |
|  | Riding school | 30.13 ± 1.58 |  |  |
|  | Carriage pulling | 30.07 ± 1.66 |  |  |
| FB01 | General training | 84.32 ± 3.34 | 2.75 | 0.04 |
|  | No working task | 83.33 ± 3.28 |  |  |
|  | Classical dressage | 83.78 ± 3.16 |  |  |
|  | Riding school | 81.75 ± 3.19 |  |  |
|  | Carriage pulling | 80.68 ± 4.54 |  |  |
| FB02 | General training | 121.00 ± 5.40 | 3.75 | 0.008 |
|  | No working task | 120.54 ± 5.22 |  |  |
|  | Classical dressage | 121.33 ± 4.27 |  |  |
|  | Riding school | 119.38 ± 6.41 |  |  |
|  | Carriage pulling | 114.93 ± 7.46 |  |  |
| FB03 | General training | 188.82 ± 6.11 | 1.31 | 0.28 |
|  | No working task | 186.21 ± 6.04 |  |  |
|  | Classical dressage | 189.14 ± 6.26 |  |  |
|  | Riding school | 187.56 ± 8.17 |  |  |
|  | Carriage pulling | 184.96 ± 6.42 |  |  |
| FB07 | General training | 26.91 ± 3.01 | 2.96 | 0.03 |
|  | No working task | 28.00 ± 2.92 |  |  |
|  | Classical dressage | 27.03 ± 2.89 |  |  |
|  | Riding school | 29.69 ± 3.35 |  |  |
|  | Carriage pulling | 29.37 ± 2.22 |  |  |
| FB08 | General training | 27.91 ± 2.83 | 1.30 | 0.28 |
|  | No working task | 26.96 ± 3.49 |  |  |
|  | Classical dressage | 26.36 ± 2.92 |  |  |
|  | Riding school | 26.44 ± 2.11 |  |  |
|  | Carriage pulling | 25.66 ± 2.40 |  |  |
| FB12R | General training | 21.55 ± 3.51 | 1.39 | 0.25 |
|  | No working task | 20.46 ± 0.84 |  |  |
|  | Classical dressage | 20.39 ± 0.68 |  |  |
|  | Riding school | 20.38 ± 1.06 |  |  |
|  | Carriage pulling | 20.27 ± 0.77 |  |  |
| FB14 | General training | 19.32 ± 1.12 | 0.20 | 0.94 |
|  | No working task | 19.50 ± 0.80 |  |  |
|  | Classical dressage | 19.39 ± 0.56 |  |  |
|  | Riding school | 19.63 ± 0.92 |  |  |
|  | Carriage pulling | 19.50 ± 0.89 |  |  |
| FB16R | General training | 24.00 ± 0.71 | 1.25 | 0.30 |
|  | No working task | 23.21 ± 0.81 |  |  |
|  | Classical dressage | 23.44 ± 1.28 |  |  |
|  | Riding school | 23.13 ± 1.03 |  |  |
|  | Carriage pulling | 23.75 ± 1.27 |  |  |
| FB17R | General training | 13.77 ± 0.52 | 2.43 | 0.06 |
|  | No working task | 13.63 ± 0.57 |  |  |
|  | Classical dressage | 13.75 ± 1.00 |  |  |
|  | Riding school | 13.31 ± 1.10 |  |  |
|  | Carriage pulling | 13.02 ± 0.92 |  |  |
| FB18 | General training | 9.73 ± 1.10 | 2.97 | 0.03 |
|  | No working task | 9.26 ± 0.55 |  |  |
|  | Classical dressage | 9.58 ± 0.96 |  |  |
|  | Riding school | 9.44 ± 1.08 |  |  |
|  | Carriage pulling | 10.27 ± 0.91 |  |  |
| FB24 | General training | 58.05 ± 1.88 | 0.90 | 0.47 |
|  | No working task | 58.83 ± 2.33 |  |  |
|  | Classical dressage | 57.83 ± 2.26 |  |  |
|  | Riding school | 57.50 ± 2.00 |  |  |
|  | Carriage pulling | 57.36 ± 2.42 |  |  |
| FB27 | General training | 97.18 ± 2.40 | 0.49 | 0.74 |
|  | No working task | 97.92 ± 2.39 |  |  |
|  | Classical dressage | 97.17 ± 2.31 |  |  |
|  | Riding school | 98.38 ± 2.83 |  |  |
|  | Carriage pulling | 98.00 ± 3.19 |  |  |
| FB28 | General training | 54.09 ± 1.76 | 0.21 | 0.93 |
|  | No working task | 54.33 ± 2.10 |  |  |
|  | Classical dressage | 54.44 ± 2.06 |  |  |
|  | Riding school | 54.25 ± 2.04 |  |  |
|  | Carriage pulling | 54.71 ± 1.93 |  |  |
| FB32R | General training | 9.68 ± 0.60 | 4.70 | 0.002 |
|  | No working task | 9.33 ± 0.62 |  |  |
|  | Classical dressage | 9.83 ± 0.73 |  |  |
|  | Riding school | 9.75 ± 1.00 |  |  |
|  | Carriage pulling | 10.66 ± 1.30 |  |  |
| FB36 | General training | 186.55 ± 4.16 | 0.24 | 0.91 |
|  | No working task | 185.42 ± 3.42 |  |  |
|  | Classical dressage | 187.14 ± 5.02 |  |  |
|  | Riding school | 186.86 ± 5.11 |  |  |
|  | Carriage pulling | 186.46 ± 5.50 |  |  |
| Weight | General training | 501.7 ± 42.3 | 1.13 | 0.35 |
|  | No working task | 485.6 ± 24.6 |  |  |
|  | Classical dressage | 506.8 ± 33.0 |  |  |
|  | Riding school | 484.4 ± 42.7 |  |  |
|  | Carriage pulling | 495.4 ± 44.8 |  |  |
